# Multi-Level Classification of Driver Drowsiness by Simultaneous Analysis of ECG and Respiration Signals Using Deep Neural Networks

**DOI:** 10.3390/ijerph191710736

**Published:** 2022-08-29

**Authors:** Serajeddin Ebrahimian, Ali Nahvi, Masoumeh Tashakori, Hamed Salmanzadeh, Omid Mohseni, Timo Leppänen

**Affiliations:** 1 Department of Applied Physics, University of Eastern Finland, 70210 Kuopio, Finland; 2 Virtual Reality Laboratory, K. N. Toosi University of Technology, Tehran 19697-6449, Iran; 3 Diagnostic Imaging Center, Kuopio University Hospital, 70210 Kuopio, Finland; 4 Department of Industrial Engineering, K. N. Toosi University of Technology, Tehran 19697-6449, Iran; 5 Lauflabor Locomotion Lab, Institute of Sports Science, Centre for Cognitive Science, Technische Universität Darmstadt, 64283 Darmstadt, Germany; 6 School of Information Technology and Electrical Engineering, The University of Queensland, Brisbane 4072, Australia

**Keywords:** ECG, respiration, deep learning, drowsiness detection, multi-level classification

## Abstract

The high number of fatal crashes caused by driver drowsiness highlights the need for developing reliable drowsiness detection methods. An ideal driver drowsiness detection system should estimate multiple levels of drowsiness accurately without intervening in the driving task. This paper proposes a multi-level drowsiness detection system by a deep neural network-based classification system using a combination of electrocardiogram and respiration signals. The proposed method is based on a combination of convolutional neural networks (CNNs) and long short-term memory (LSTM) networks for classifying drowsiness by concurrently using heart rate variability (HRV), power spectral density of HRV, and respiration rate signal as inputs. Two models, a CNN-based model and a hybrid CNN-LSTM-based model were used for multi-level classifications. The performance of the proposed method was evaluated on experimental data collected from 30 subjects in a simulated driving environment. The performance and the results of both models are presented and compared. The best performance for both three-level and five-level drowsiness classifications was achieved by the CNN-LSTM model. The results indicate that the three-level and five-level classifications of drowsiness can be achieved with 91 and 67% accuracy, respectively.

## 1. Introduction

Drowsiness is one of the leading causes of road accidents. Driving is a complex process that needs the driver’s full attention and awareness, which can be highly affected by drowsiness. It is estimated that falling asleep at the wheel accounts for approximately 7% of road accidents and 17% of fatal crashes [1]. Even with ever-increasing progress in automated driving technologies, driver drowsiness is not negligible. Studies focused on the human factors of automated driving have shown increased levels of mental fatigue and drowsiness during automated driving [2,3]. In current automated driving technologies, based on the automation level in vehicles, the driver’s intervention might be needed in takeover situations. Thus, even in some levels of automated driving, drowsiness is still an important issue that needs to be addressed until fully autonomous driving becomes feasible.

To reduce the number of crashes caused by drowsy driving, several countermeasures have been introduced by researchers. Measures such as having sufficient sleep, reduction of the continuous driving period, and short naps after several hours of driving have long been advised by researchers to prevent driver drowsiness. Drivers’ misjudgment and overconfidence highlight the need for in-vehicle drowsiness countermeasures. There are three broad categories in in-vehicle drowsiness detection methods: physiological signals, the driver’s facial characteristics, and vehicle dynamics [4]. Among these methods, physiology-based drowsiness detection systems have been shown to yield more reliable accuracy compared to other drowsiness detection methods [4]. Physiological signals such as electroencephalography (EEG), electrooculography (EOG), electrocardiography (ECG), electromyography (EMG), respiration, galvanic skin resistance, and body temperature have all been used for drowsiness detection and have shown high accuracy [5,6]. However, the use of these methods is still limited due to the intrusive nature of signal acquisition methods. Despite efforts to enable non-intrusive measurement of EEG, EMG, and EOG signals, acquisition methods for these signals still require direct contact and permanent placement on the driver’s body causing discomfort. Fortunately, ECG can be achieved by embedding measurement sensors in the steering wheel [7], driver’s seat [8], and small wearables such as armbands [9], causing the least discomfort to the driver. On the other hand, contactless measurement of the respiration signal is achievable through non-contact methods in driving conditions [10,11,12,13,14]. Thus, ECG and respiration signals can be considered good choices for drowsiness measurement. 

ECG signals are used in a vast variety of applications related to the estimation of a driver’s state. In previous studies, ECG was used to examine mental workload [15], emotion [16], stress [17], and drowsiness [18]. The main feature of the ECG signal used for drowsiness detection is the heart rate variability (HRV) as it corresponds to the autonomic neural system (ANS) regulation. ANS activity alters during stress, extreme fatigue, and drowsiness episodes [19]. The HRV signal is defined as the variation of time intervals between two consecutive heartbeats and it can be analyzed in both time and frequency domains. The common frequency domain analysis of HRV is the power spectral analysis comprising four main components: ultra-low frequency (0.0–0.0033 Hz), very low frequency (0.0033–0.04 Hz), low frequency (0.04–0.15 Hz), and high frequency (0.15–0.4 Hz). The low-frequency (LF) component of the HRV power spectrum is influenced by parasympathetic and sympathetic activity. The high-frequency (HF) component is influenced by parasympathetic activity. The ratio of LF to HF components is considered a description of sympathovagal balance [19]. The LF to HF ratio decreases progressively as drowsiness increases [4].

Various analyses of the HRV signal have been used for drowsiness detection. Patel et al. [20] used the frequency component of the HRV signal derived from the power spectrum density as the input to an artificial neural network. Some studies used a mixture of the time and frequency parameters of HRV [21,22]. Gang li et al. [23] and Babaeian et al. [24] used the wavelet transform and short-time Fourier transform for analyzing the time-frequency components of the HRV. Some studies focused on fusing HRV parameters with other physiological signals. Vicente et al. [19] used HRV and respiration signals for two-level drowsiness classification and Al-Libawy et al. [25] combined HRV measures with driver’s skin temperature and skin conductance to detect drowsiness.

Respiration is also affected by the ANS and is altered during drowsiness. In previous works, it has been shown that respiration undergoes notable changes from wakefulness to drowsiness as the respiration rate decreases at higher levels of drowsiness [13,26,27]. Studies focused on respiration analysis for drowsiness detection used respiration rate or respiration rate variability [14,28,29]. Furthermore, some studies focused on deriving respiration parameters directly from the HRV signal [30,31] or using respiration in combination with other physiological signals [32,33]. Thus, the availability of contactless respiration acquisition methods makes it suitable for monitoring drowsiness. Thermal imaging was also used in several studies as a contactless device to monitor physiological parameters such as the respiration and facial temperature pattern of the drivers during drowsiness and showed promising results [13,14,34,35]. Accordingly, thermal imaging for monitoring respiration parameters during driving has been proven as a reliable method. 

Drowsiness classification has been performed with various methods, ranging from state-of-the-art machine learning and deep learning methods to traditional statistical methods. Machine learning methods such as support vector machines (SVM) [36], artificial neural networks (ANN) [20], K-nearest neighbor (KNN) [37], ensemble logistic regression [24], and decision trees [38] have been used for drowsiness detection. There are several studies for drowsiness detection that use deep learning methods such as convolutional neural networks (CNNs) [39], recurrent neural networks (RNNs) [40], deep belief networks (DBNs) [41], and hybrid networks [42,43,44]. Although traditional machine learning methods are not inferior to the deep learning methods, the ability of deep learning methods to fuse the feature extraction, feature selection, and model construction steps of a classification problem has made these methods favorable for optimal classification and the capture of the non-linear correlations across data modalities. 

Most drowsiness studies focus on whether the driver is wakeful or sleepy rather than investigate the multi-level transition from wakefulness to the last stage of drowsiness. This transition can be divided into multiple stages and each stage can be assigned a scoring level based on different drowsiness scoring methods. For instance, the Karolinska Sleep Scaling (KSS) method suggests nine levels of drowsiness from extremely alert to extremely sleepy [45], while the Stanford Sleepiness Scale (SSS) and the Observer Rating of Drowsiness (ORD) methods made this classification within seven and five levels, respectively [46,47]. Usually, drowsiness detection studies focus on choosing a threshold to reduce this multi-level classification to binary states, i.e., wakefulness and sleepiness. This binary classification increases the detection accuracy; however, it may detect drowsiness too late, resulting in accidents, particularly at higher levels of drowsiness. Consequently, detecting multiple levels of drowsiness would help to assign appropriate countermeasures before the driver becomes extremely drowsy. Proper drowsiness detection warning systems could then be utilized to prevent accidents with a long lead time.

This work is concerned with proposing a multi-level driver drowsiness detection method based on simultaneous analysis of respiration and ECG data. Even though some parameters of respiration signals are extractable from ECG signals, we recorded each signal independently. This source independency would reduce errors and false detections. In order to achieve a high accuracy and optimal multi-level classification, deep neural networks (DNNs) were used in this work. In this study, drowsiness was categorized into five levels: wakeful, slightly drowsy, moderately drowsy, very drowsy, and extremely drowsy. Two multi-level drowsiness classifications based on three levels and five levels were studied. The experiments were conducted on 30 subjects to evaluate the proposed drowsiness detection system. 

This work contributes to the field of driver drowsiness detection on three grounds. First, it targets multi-level classification of drowsiness with three and five levels instead of binary classification using physiological data of ECG and respiration. Detecting drowsiness at multiple levels would help to assign appropriate countermeasures before the driver becomes extremely drowsy. Second, it proposes a DNN architecture based on the combination of CNN and long short-term memory (LSTM) networks for the classification problem to achieve higher detection accuracies. Third, it uses two physiological signals one of which is acquired through imaging techniques and the other can be recorded non-intrusively by embedding into the vehicle steering wheel or through small wearables, thereby it is applicable for industrial use. 

The structure of the subsequent sections of this paper is as follows: Section 2 covers the details of the experimental design and methodology. The experimental results are presented in Section 3. Finally, the discussion of the results is presented in Section 4 and the paper is concluded in Section 5.

## 2. Materials and Methods

In this paper, two multi-level (i.e., 3-level and 5-level) classification schemes were studied for detecting drowsiness. Driving tests were performed in a driving simulator to study variations in respiration and HRV related to the drivers’ levels of drowsiness. Facial thermal images and ECG signals were used to extract respiration and HRV signals. Two night-vision cameras were used to monitor drivers’ conditions and estimate their drowsiness level by three human observers. Signals were pre-processed and prepared before applying the classification. Finally, two DNN models were utilized for multi-level classification based on CNN and LSTM networks.

### 2.1. Participants

The driving tests were conducted for 30 male subjects with a mean (±standard deviation, SD) age of 31 (±2.6) years. The mean (±SD) body mass index (BMI) of the participants was 27.2 (±2.3) kg/m^2^. All participants were volunteers and they were recruited through the K. N. Toosi University of Technology’s social media channels. All subjects had driving licenses and a minimum of 2 years’ driving experience. They were asked to have regular sleep and refrain from using stimulants and caffeinated substances for 48 h before the tests. Neither of the subjects was addicted to drugs or alcohol and all were non-smokers. Moreover, the subjects reported no previous history of diabetes, respiratory, cardiovascular, or neurological diseases. The subjects signed consent forms prior to the test and observed the experimental protocol. The study protocol was approved by the K. N. Toosi University of Technology in accordance with the Declaration of Helsinki.

### 2.2. Test equipment

**Figure 1:**
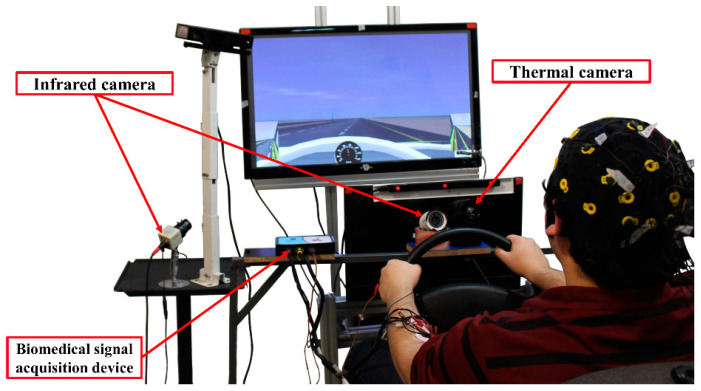
Nasir Semi 003TM driving simulator and placement of data acquisition devices. In this study, only data measured from thermal camera and ECG signals were used.

The tests were performed in a driving simulator in a dark and controlled room to ensure drivers’ safety and decrease measurement errors. The subjects drove in a driving simulator built at K. N. Toosi University of Technology consisting of a set of pedals, steering wheels, a gear stick, and a monitor (Figure 1). The simulator’s software simulated the dynamic model of a 14 degree-of-freedom vehicle solved in real-time and a graphical rendering engine operating at a frame rate of 30 Hz. Two night-vision cameras were installed to monitor the driver’s drowsiness. A thermal camera with a sensitivity of 50 m Kelvin, a resolution of 640 × 480, and a frame rate of 7.5 frames per second was used to record thermal images of the driver. A biomedical signal acquisition device was installed to record ECG data of the drivers through electrodes attached to the driver. Data acquisition devices were all controlled through a central computer, and the data was synchronized based on a trigger signal issued at the onset of data recording. 

#### 2.2.1. ECG

**Figure 2:**
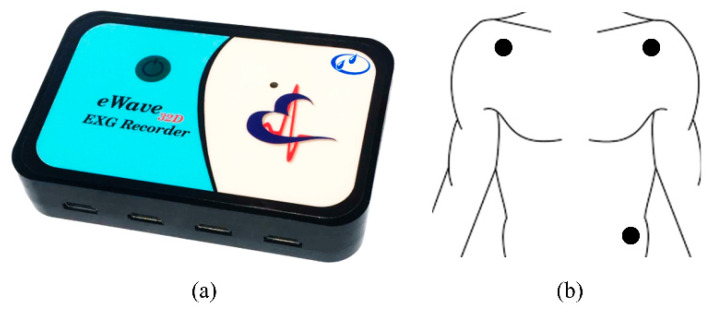
(**a**) Biomedical data acquisition device; (**b**) ECG lead placement.

The ECG signal was recorded during driving by a portable digital recording system. The portable data acquisition system was eWave32D from ScienceBeamTM Company with a sampling rate of 1 kHz. The electrodes were disposable Ag/AgCl Skintact F WA10C with rectangular leads. The skin was rubbed with isopropyl alcohol before attachment to the electrodes. The data acquisition device and electrode placements are shown in Figure 2.

The ECG signal was filtered and processed to extract the HRV signal. First, the Grubbs outlier detection method [48] was used to detect and remove the outliers and movement artifacts from the signal. Then, a fourth-order Butterworth band-pass filter with a bandpass frequency of 2–40 Hz was used to remove baseline wander, muscle movement noise, powerline interference, and high-frequency noise. The Pan–Tompkins method [49] was used to detect R-peaks from the ECG signals. Then, the HRV signal was formed by sequencing the time intervals between each two consecutive R-peaks. RR intervals with a relative difference greater than 30% of the mean of the most recent four previous intervals were considered outliers [50]. The fast Fourier transform was used to obtain the power spectral density (PSD) of the HRV signal. Then the frequency range of the calculated PSD was capped to the frequency range corresponding to the changes in drowsiness (0.04 Hz to 0.4 Hz).

#### 2.2.2. Respiration 

**Figure 3:**
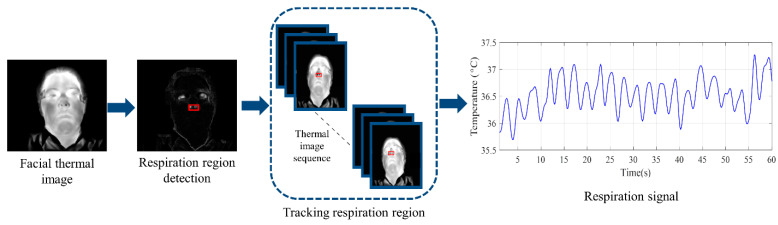
Respiration signal extraction procedure: First, the respiration region was extracted by cumulating temporal changes in the first few frames of the thermal sequence. Then, it was tracked in thermal image sequences, and finally, the signal was formed by calculating the mean temperature of the respiration region in each frame.

A thermal camera was used to monitor the driver’s respiration. The respiration region—the nostrils and the region beneath—was localized by using spatial continuity in the thermal image sequences and cumulating temporal changes in the first few frames of the thermal sequence [14]. After localizing the respiration region, the region was tracked in subsequent frames by a spatiotemporal context learning tracker [51]. In this method, the tracking takes place using a confidence map that represents an estimate of the probable location of the region and maximizes the value of the region [51]. The mean of the pixels in the tracked region was considered the respiration signal and was calculated for each frame. The respiration signal extraction in this work was based on [14]. The procedure for extracting the respiration signal from thermal images is shown in Figure 3.

After forming the respiration signal, the signal was filtered and processed. The Grubbs outlier detection method [48] was used to remove outliers and minor driver movement artifacts. Next, a fourth-order Butterworth low-pass filter with a cutoff frequency of 0.6 Hz was used to reduce high-frequency noises. Afterward, a synchrosqueezing transform was used to extract time-frequency components of the respiration signal as instantaneous respiration rate [52]. The respiration rate signal was downsampled to 1 Hz before using for classification in order to reduce the size of the input signals for classification. 

### 2.3. Experimental protocol

**Figure 4:**
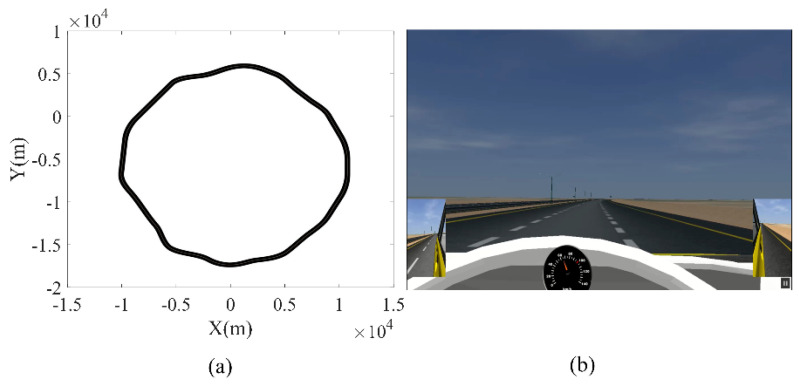
(**a**) Quasi-circular map of the three-lane highway with an average radius of 10 km; (**b**) graphical view of the road.

All subjects had similar test conditions. Before the test, each driver had a 15 min drill session to become familiar with the simulator. The tests were conducted in two 80 min sessions between 1:00 p.m. to 2:30 p.m. after having lunch and between 3:30 p.m. to 5:00 p.m. The drivers were asked to drive on a three-lane highway with a quasi-circular path, keep on the middle lane, and drive as fast as 70 km/h to 90 km/h. If the subject failed to control the vehicle in the middle lane and diverged to the guardrail or the road’s shoulder three times, the test session was terminated. The highway was designed with no obstacles or vehicles along the way to minimize distractions. The specific design of the road parameters (i.e., monotonic road, no other vehicles) made the driver more prone to drowsiness. Furthermore, drowsy drivers departed from the middle lane because of the existence of soft turns in the road. The road map and the road scene are shown in Figure 4.

### 2.4. Drowsiness Scale

The Observer Rating of Drowsiness (ORD) [47] was used to label the level of drowsiness. The ORD method was based on the mean assessments of three trained human observers without using any physiological signals. Observers used the driver’s body movement and facial changes to score the drowsiness level from 1 to 5 corresponding to wakeful, slightly drowsy, moderately drowsy, very drowsy, and extremely drowsy. Each observer rated drowsiness separately and the mean of the scores was used as the final score. Each observer filled the ORD behavior and mannerism checklist (Appendix A) for each 1 min segment of driving. Based on the severity of facial and behavioral features of the driver in the checklist and definitions of the drowsiness levels in the ORD method, one of the drowsiness levels was assigned to each segment. 

### 2.5. Deep Neural Networks

**Figure 5:**
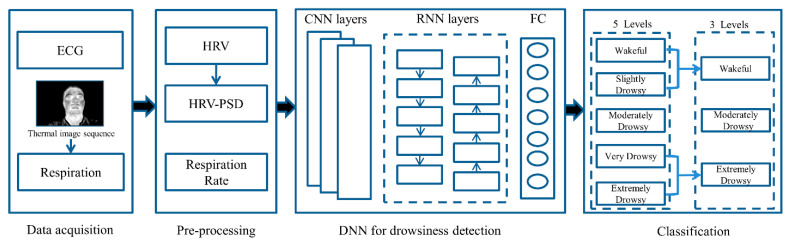
Overall flowchart of the proposed method for 3-level and 5-level classifications. ECG: electrocardiogram, CNN: convolutional neural network, RNN: recurrent neural networks, FC: fully connected.

The pre-processed data obtained from the experiments were used to classify different levels of drowsiness using DNNs and classifiers were selected as a combination of CNN, RNN, and fully connected (FC) layers. Three input signals were considered for the classifiers: HRV, power spectral density of HRV (HRV-PSD), and the respiration rate (RR). The classification was conducted individually for two different outcome configurations: a 3-level classification and a 5-level classification. Classification levels were based on drowsiness levels in the ORD method. In the 3-level classification, ORD < 3 was considered as wakeful, ORD = 3 as moderately drowsy, and ORD > 3 as extremely drowsy. For the 5-level classification, all 5 levels of ORD scores were considered for the classification. The overall pipeline of the process is shown in Figure 5.

The input to the neural networks was the pre-processed data split into 2 min epochs. Epochs were formed with 60% overlap. The dataset was then normalized using z-score normalization by subtracting the data from its mean and dividing by the standard deviation of the training data. Across all data, 80% were randomly selected as the training set and the remaining 20% as the test set for adopting 5-fold cross-validation.

#### Model Architecture

Drowsiness level classification was carried out with the combined CNN and LSTM. The CNN part of the network was utilized to extract local invariant features, while the LSTM was utilized to consider temporal distribution features.

**Figure 6:**
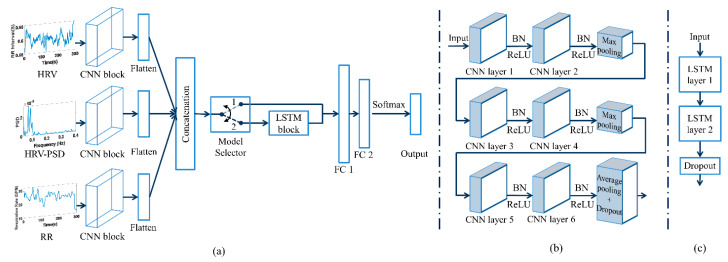
(**a**) The model architecture of the CNN and the hybrid CNN-RNN models. The model selector block is a hypothetical block to show the difference between the two models: state 1 shows the CNN model and state 2 shows the CNN-LSTM model; (**b**) details of the inner layers in the CNN blocks; (**c**) details of the LSTM block. CNN: convolutional neural network, HRV: heart rate variability, PSD: power spectral density, RR: respiration rate, LSTM: long short-term memory, FC: fully connected, BN: batch normalization, and ReLU: rectified linear unit.

Two model architectures were used and the performances of the classifiers were compared. The first model comprised a CNN block and fully connected layers. The second model was a hybrid CNN-LSTM model in which an LSTM block was added to the first model. The models were implemented in Python 3.6 using Keras API with TensorFlow backend. As shown in Figure 6a, both models contain 3 identical parallel CNN blocks for each of the 3 input signals. The information from the parallel CNN blocks was then flattened and concatenated before passing it to the fully connected layers or LSTM block, depending on the model type. The CNN block consisted of six 1D convolution layers, two max-pooling layers, and a global average pooling layer as shown in Figure 6b. Each 1D convolution was followed by batch normalization (BN) and a rectified linear unit (ReLU) activation function. The pooling layers were placed after two CNN layers. The last 1D convolution was followed by a global average pooling layer and a dropout layer. The LSTM block in the hybrid CNN-LSTM model consisted two bidirectional long short-term memory layers Figure 6c. In both models, the information from two dense layers was fed to a 3- or 5-way softmax layer to produce the output sequence of drowsiness level.

The optimization of hyperparameters was set to achieve the best performance. The grid search method was used to adjust the hyperparameters of models. For simplicity, only some of the model hyperparameters were selected through grid search (Table 1) and the rest determined by the authors, based on trial and error and existing literature [44,53,54,55,56,57]. The weights and the hyperparameters of the trained model resulting in the lowest loss of the training process were stored and selected as the final values. Table 1 shows the optimized values of hyperparameters for both models.

For configuring the models, the number of filters used by the first two sets of convolution layers (i.e., CNN layers 1 and 2) was selected to be the same, and the hyperparameters of the first layer were selected by a grid search. The other two subsequent pairs of convolution layers were chosen to have twice the number of filters used in the previous layers with the same length. Thus, each two adjacent CNN layers placed in series (Figure 6b) have the same number of filters and twice the number of their predecessor layers (i.e., 128 for CNN layers 3 and 4 and 256 for CNN layers 5 and 6). The number of channels of the batch normalization layer was chosen to be the same as the number of filters selected in the convolution layer (i.e., 64). The pooling layers had a pool size of 2. A stride of one was used for the convolution layers and a stride of two for the pooling layers. For the LSTM layers, the number of cells in the first layer was selected by grid search and the subsequent layer had half the cells of the first layer (i.e., 128). In the fully connected layers, the size of the first layer was equal to the size of the previous layer—the last CNN layer for the first model and the last LSTM layer for the second model (i.e., 256 for CNN model and 128 for CNN-LSTM model). The subsequent dense layer was half the size of the previous layer.

The number of iterations for the training process was 300. The cross-entropy loss function was selected as the cost function, and the Adam optimizer was exploited as the optimization method. Class weights were used to compensate for imbalances in the dataset. Early stopping was also used to halt the training process when there was no reduction in the loss function for the validation data. The final classification performance was evaluated on the test dataset in terms of the average classification accuracy, sensitivity, specificity, and precision. 

## 3. Results

The performance of both models for all combinations of input signals is presented for three-level (Table 2) and five-level (Table 3) classifications. As expected, the best performance was achieved with using all signals as an input. Using all input signals, the CNN model achieved 84 and 62% accuracy for the three-level and the five-level drowsiness classifications, respectively. The CNN-LSTM model achieved 91 and 67% accuracy for these two levels. In both cases, the best performances were achieved by the CNN-LSTM model.

Despite the good accuracy obtained using all the three input signals together, single or double usage of the inputs did not yield satisfactory results. According to Table 2 and Table 3, the results for single usage of the signals were not adequate with an exception of 81% accuracy using the RR signal in the three-level classification by the CNN-LSTM network. As for the pairwise combination of the inputs, the three-level classification by HRV and RR was better than the other combinations. The other two pairwise combinations showed nearly similar results. Therefore, the results indicate that the HRV and RR signals can be considered the main factors for classification. 

**Figure 7:**
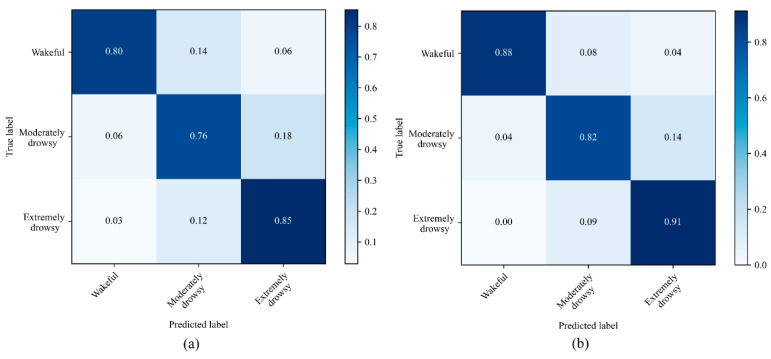
Normalized confusion matrices for the 3-level drowsiness classification in the (**a**) CNN model and the (**b**) CNN-LSTM model. For this classification, we considered ORD < 3 as wakeful, ORD = 3 as moderately drowsy, and ORD > 3 as extremely drowsy. ORD: observer rating of drowsiness.

**Figure 8:**
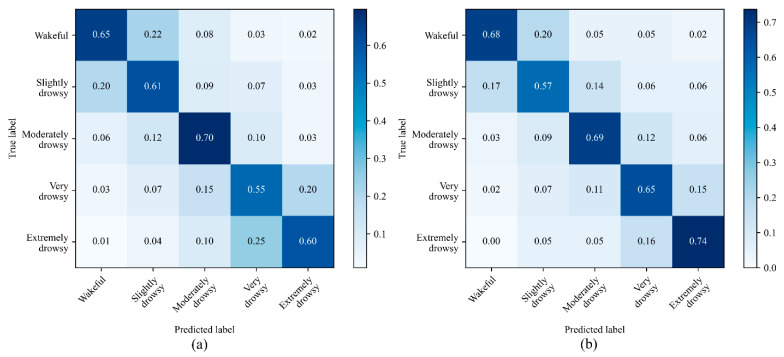
Normalized confusion matrices for 5-level drowsiness detection in the (**a**) CNN model and the (**b**) CNN-LSTM model.

Figure 7 and Figure 8 show the normalized confusion matrices for the three-level and the five-level classifications using CNN and CNN-LSTM models with all signals used as an input. For three drowsiness levels, the sensitivities were 80% for wake, 76% for moderately drowsy, and 88% for extremely drowsy in the CNN model (Figure 7a). In the CNN-LSTM model, the sensitivities were 88% for wake, 82% for moderately drowsy, and 91% for extremely drowsy (Figure 7b). The sensitivities for five-level drowsiness detection were 65% for wake, 61% for slightly drowsy, 70% for moderately drowsy, 55% for very drowsy, and 60% for extremely drowsy in the CNN model (Figure 8a). In the CNN-LSTM model, the sensitivities were 68% for wake, 57% for slightly drowsy, 69% for moderately drowsy, 65% for very drowsy, and 74% for extremely drowsy (Figure 8b).

The CNN-LSTM model resulted in the highest accuracy and sensitivity in the three-level classification with the combined usage of HRV, HRV-PSD, and respiration rate signals. This model achieved an accuracy of 91%, sensitivity of 87%, specificity of 92%, and precision of 87%. For five-level classification, on the other hand, the results for both models were not sufficient enough for detecting all five levels of drowsiness. For example, the CNN-LSTM resulted in an accuracy of 67%, which is 24% less than the accuracy achieved by the three-level classification.

## 4. Discussion

Both ECG and respiration signals can be acquired non-intrusively without interfering with the driving task. In this paper, the respiration signal was extracted from the thermal imaging sequence, while the ECG signal was recorded with three leads attached to the driver’s chest. The ECG signals can also be recorded using sensors embedded in the steering wheel, seatbelt, or wristband. The results obtained in this work can be further strengthened by combining the two signals with other non-intrusive physiological signals such as EMG, facial temperature, or photoplethysmography (PPG).

A summary of the recent research works that performed multi-level drowsiness classification using physiological signals is presented in Table 4. For a fair comparison and to avoid any possible biases, this table only summarizes the studies that classified multiple levels of drowsiness using physiological data. Barua et al. [58] employed a support vector machine for the three-level classification of drowsiness. A combination of features from EEG and EOG signals were used for classification and the results were further strengthened by adding contextual data including awake time, driving condition, and lighting condition. Persson et al. used a combination of 24 HRV-derived features using a random forest classifier for three-level classification in a large dataset comprising three separately recorded datasets [22]. Arefnezhad et al. utilized scalograms of ECG signals using CNN for three-level classification [59]. In comparison, our presented CNN-LSTM method outperformed these methods in three-level drowsiness detection (91 vs. 79% [58], 91 vs. 64% [22], and 91 vs. 79% [59]). Lemkaddem et al. employed an SVM with a combination of PPG signal and camera-derived EOG parameters [60]. The results were comparable to those of our work in the three-level classification (91 vs. 89% [56]). Hultman et al. used a combination of raw EEG and EOG signals as inputs of a CNN-LSTM network for a five-level classification of drowsiness in an extensively large dataset comprising 12 separate datasets from different published works [53]. In comparison, the results obtained from the five-level classification in our work outperformed that study by 21%. One of the possible causes related to the poor performance achieved in that work could be the variations between different datasets. Jeon et al. used raw EEG signals as input of a deep spatiotemporal convolutional bidirectional LSTM network for five-level drowsiness detection [54]. Our proposed method slightly underperformed (2%) compared to that work, which could be due to the more sophisticated designs of DNN. 

The outcome of the five-level classification was not as accurate as that of the three-level classification. The confusion matrices in Figure 8 show that most misclassifications occurred between wakefulness and slightly drowsy states and also between very drowsy and extremely drowsy states. These misclassifications show that despite behavioral differences between sequential levels of the ORD method, the selected physiological signals do not differ so much between these adjacent levels. As shown in the confusion matrices of the three-level classification (Figure 7), bundling both wakefulness and slightly drowsy states as wakefulness, and very drowsy and extremely drowsy states as extremely drowsy, classification results improved significantly by 24%. The ORD method is based on the facial and behavioral changes of the drivers. Some subjects may not exhibit significant changes in facial and behavioral features in adjacent levels when the pace of drowsiness level change is high. This limitation does not exist in the three-level classification as there is a time gap between adjacent levels. Furthermore, the low accuracy of the five-level classification can originate from the mechanical inertia of the cardiorespiratory system that causes slow changes at different levels of drowsiness. In other words, the time constant of mechanical features such as respiration is longer than that of EEG signals and exhibits latencies. Therefore, a non-mechanical signal such as EEG could be more suitable for detecting five distinct levels of drowsiness, though the EEG measurement device may lead to driver’s discomfort and is prone to disturbances. 

There are several drowsiness evaluation methods in the literature, such as the ORD, KSS, blink duration, and reaction time tests. Although the KSS method is well-respected and used in many studies, it has major drawbacks. The KSS method is self-explanatory and these self-introspections interfere with the natural progress of driver drowsiness. The KSS method is also affected by the driver’s subjective interpretation of the level of drowsiness, as the subject’s self-evaluation does not always reflect the accurate level of drowsiness. Furthermore, KSS scoring is performed every 5 min, while the time constant of drowsiness dynamics decreases at higher levels of drowsiness requiring shorter labeling epochs. Blink duration measurement devices are not accurate at the early stages of drowsiness since the blink duration would not change in the early stages. These methods perform well only at higher levels of drowsiness where changes in blink duration are significant. Reaction time tests are difficult to administer during driving as it requires repetitions, causing distraction and discomfort to the driver. The ORD method used in this paper has certain advantages, such as non-intrusiveness, short time intervals of scoring, and strict multi-aspect scoring rules. However, a small percentage of subjects may not exhibit different facial expressions in adjacent levels of drowsiness when the rate of drowsiness progress is rapid. This fact could be one of the reasons for the lower accuracy of the five-level classification compared with that of the three-level classification.

Driver drowsiness detection must be robust and accurate. Partial signal loss and environmental disturbances may vastly affect the performance of a system that works solely based on physiological signal analysis. One of the best solutions to compensate for these shortcomings is to simultaneously use sensors from other sources. Previous studies have shown that vehicle-based and facial measurements are rather accurate in multi-level drowsiness classifications [44,61,62], but they can be affected by the road geometry and lighting conditions. Fusing physiological features with vehicle dynamics and the drivers’ facial features improve the classification accuracy and compensate for the temporary loss of signals.

There are many possible combinations of layer types, hyperparameters, activation functions, and optimization functions in a DNN model. Not all possible solutions have been investigated in this paper. For some parameters such as activation functions and optimization methods, selections were made based on common choices in the literature. To determine the number of architecture layers, we first started with one pair of CNN layers and then increased it by adding other pairs to raise the complexity of the network until the accuracy was saturated. We opted for a similar procedure for the single LSTM and FC layers. As for the model hyperparameters, three identical CNN blocks for each input were considered and only a selected number of hyperparameters were investigated for all parts of the model. For example, the number of filters and the kernel size were chosen by applying a grid search. The rest of the parameters were determined by the authors based on trial and error and existing literature [44,53,54,55,56,57].

Individual differences between subjects can affect the performance of drowsiness detection methods. Although there are general trends in physiological processes during drowsiness, these changes can be more or less sensitive for some subjects. Using a user-specific method for detecting a driver’s drowsiness can vastly improve the performance of these methods in real-world driving. Deep-learning methods similar to the one used in this paper can enhance classification accuracy by taking data from a specific driver to retrain the model with a new batch of data.

Several research works can be investigated to extend and enhance the methodology used in this work. In this work, an identical CNN block was used for all inputs. The effects of non-identical networks for each separate input can be further studied in future works. Other types of DNNs such as the gated recurrent networks (GRUs) can be incorporated to make the model less computationally expensive for real-driving conditions. The respiration extraction in this work was based on temporal changes in the facial patterns. One can train a DNN for faster and more precise detection of the respiration region. The method presented in this paper for normal subjects could also be extended for detecting drowsiness in drivers with sleep disorders, respiratory, or heart conditions. 

## 5. Conclusions

The main objective of this work was to develop a method to classify multiple levels of drowsiness using respiration and ECG signals. DNN-based models with different combinations of cardiorespiratory features were used. The results from the CNN-LSTM model showed 91 and 67% accuracy in the three- and five-level classifications, respectively. It was shown that the accuracy of the drowsiness level estimations was higher than most other physiological signal-based methods available in the literature. Moreover, the potential availability of non-intrusive built-in sensors in the vehicle for both respiration and ECG signals suggests that the developed algorithms can be employed for non-intrusive and precise multi-level drowsiness classification with little interference to the driver. 

## Figures and Tables

**Table 1:** Optimized hyperparameters applied to the models.

Hyperparameter	Search Space	Model 1 (CNN)	Model 2 (CNN-LSTM)
Number of filters in the first CNN layer	64, 128, 256	64	64
Filter length of the first CNN layer	3–10	5	5
Number of cells in the first LSTM layer	64, 128, 256	-	256
Batch size	32, 64, 128	64	32
Learning rate	0.001–0.00001	0.0001	0.0001

CNN: convolutional neural network, LSTM: long short-term memory.

**Table 2:** Performance of the CNN and the CNN-LSTM models for the three-level drowsiness classifications for different combinations of input signals.

Model Name	Input Signal	Accuracy (%)	Sensitivity (%)	Specificity (%)	Precision (%)
**CNN**	HRV	55	58	59	56
	HRV-PSD	63	61	61	59
	RR	66	62	61	65
	HRV + HRV-PSD	65	63	60	62
	HRV + RR	72	67	71	70
	HRV-PSD + RR	68	64	65	65
	**All inputs**	**84**	**80**	**88**	**80**
**CNN-LSTM**	HRV	72	65	71	61
	HRV-PSD	73	65	72	64
	RR	81	76	83	74
	HRV + HRV-PSD	78	71	81	70
	HRV + RR	82	77	84	80
	HRV-PSD + RR	77	71	79	70
	**All inputs**	**91**	**87**	**92**	**87**

HRV: heart rate variability, HRV-PSD: power spectral density of HRV, RR: respiration rate.

**Table 3:** Performance of the CNN and CNN-LSTM models for the five-level drowsiness classifications for different combinations of input signals.

Model Name	Input Signal	Accuracy (%)	Sensitivity (%)	Specificity (%)	Precision (%)
**CNN**	HRV	51	50	48	51
	HRV-PSD	52	53	54	52
	RR	55	59	56	52
	HRV + HRV-PSD	57	56	56	52
	HRV + RR	59	60	61	56
	HRV-PSD + RR	57	59	52	56
	**All inputs**	**64**	**62**	**67**	**62**
**CNN-LSTM**	HRV	54	50	51	50
	HRV-PSD	58	56	63	54
	RR	60	60	65	63
	HRV + HRV-PSD	61	60	61	59
	HRV + RR	63	62	62	63
	HRV-PSD + RR	60	55	62	53
	**All inputs**	**67**	**67**	**70**	**66**

HRV: heart rate variability, HRV-PSD: power spectral density of HRV, RR: respiration rate.

**Table 4:** Accuracy comparison of the proposed method with other multi-level drowsiness classification methods based on physiological signals.

Study	Method	Physiological Signal	Variable	Accuracy
Barua et al., 2018 [58]	Support vector machine	EEG and EOG	EEG-PSD, blink duration, and contextual analysis	79% (3-level)
Lemkaddem et al., 2018 [60]	Support vector machine	PPG	HRV and PERCLOS	89% (3-level)
Persson et al., 2020 [22]	Random forest classifier	ECG	HRV	64% (3-level)
Arefnezhad et al., 2022 [59]	CNN	ECG	Scalogram of ECG	79% (3-level)
Hultman et al., 2021 [53]	CNN-LSTM	ECG and EOG	Pre-processed ECG and EOG signals	46% (5-level)
Jeon et al., 2019 [54]	Deep spatiotemporal convolutional bidirectional LSTM network (DSTCLN)	EEG	Pre-processed EEG signal	69% (5-level)
**This paper**	**CNN-LSTM**	**ECG and respiration**	**HRV, HRV-PSD, and respiration rate**	**91% (3-level)** **67% (5-level)**

EEG: electroencephalogram, EOG: electrooculogram, PPG: photoplethysmography, ECG: electrocardiogram, EEG-PSD: power spectral density of EEG, HRV: heart rate variability, PERCLOS: percentage of eyelid closure over the pupil over time, HRV-PSD: power spectral density of HRV, CNN: convolutional neural networks, LSTM: long short-term memory.

## Data Availability

The data used in this work is not available for public use due to privacy policy.

## Appendix A

**Descriptions of five levels of drowsiness in ORD method:**

1. **Not Drowsy:** A driver who is not drowsy while driving will exhibit behaviors such that the appearance of alertness will be present. For example, normal facial tone, normal fast eye blinks, and short ordinary glances may be observed. Occasional body movements and gestures may occur [47].
2. **Slightly Drowsy:** A driver who is slightly drowsy while driving may not look as sharp or alert as a driver who is not drowsy. Glances may be a little longer and eye blinks may not be as fast. Nevertheless, the driver is still sufficiently alert to be able to drive [47].
3. **Moderately Drowsy:** As a driver becomes moderately drowsy, various behaviors may be exhibited. These behaviors, called mannerisms, may include rubbing the face or eyes, scratching, facial contortions, and moving restlessly in the seat, among others. These actions can be thought of as countermeasures to drowsiness. They occur during the intermediate stages of drowsiness. Not all individuals exhibit mannerisms during intermediate stages. Some individuals appear more subdued, they may have slower closures, their facial tone may decrease, they may have a glassy-eyed appearance, and they may stare at a fixed position [47].
4. **Very Drowsy:** As a driver becomes very drowsy, eyelid closures of 2 to 3 s or longer usually occur. This is often accompanied by a rolling upward or sideways movement of the eyes themselves. The individual may also appear not to be focusing the eyes properly or may exhibit a cross-eyed (lack of proper vergence) look. Facial tone will probably have decreased. Very drowsy drivers may also exhibit a lack of apparent activity, and there may be large isolated (or punctuating) movements, such as providing a large correction to steering or reorienting the head from a leaning or tilted position [47].
5. **Extremely Drowsy:** Drivers who are extremely drowsy are falling asleep and usually exhibit prolonged eyelid closures (4 s or more) and similar prolonged periods of lack of activity. There may be large punctuated movements as they transition in and out of intervals of dozing [47].

**Table A1:** ORD Behavior and Mannerism Checklist [47].

		None	Minor	Moderate	Extreme
**Eyes/Eyebrows**	Rubbing/scratching				
	Blank/fixed stare				
	Squinting				
	Excessive/hard blinking				
	Slow closure				
	Unfocused rolling				
	Glassy/glazed				
	Raise/open wide				
	Lower/scowl				
**Body**	Slumping/slouching/leaning				
	Sighing				
	Stretching				
	Bodyrolling/slack muscle tone				
	Body position change (restlessness)				
**Mouth**	Yawning				
	Biting/licking lips				
	Tongue motion				
**Face**	Rubbing/holding				
	Facial contortions				
	Slack muscle tone				
**Neck/Head**	Hair: scratching/straightening				
	Rubbing/holding				
	Head learning (back or side, unsupported)				
	Head position change				
	Head nodding/drooping				
